# Kaiserling’s Method for the Preservation of Specimens with Their Natural Colors

**Published:** 1897-09

**Authors:** 


### Kaiserling's Method for the Preservation of Specimens with their Natural Colors.

The above method was referred to by Dr. W. F. Whitney, at the March meeting of the Boston Society of Medical Sciences.

Slices of organs from 3 to 5 ctm. thick are placed from 3 to 5 days in:

1. Formaldehyd..................................200 c.c.
   Water.............................................1000 c.c.
   Nitrate of potash.............................15 gme.
   Acetate of potash.............................30 gme.

They are then removed, the fluid allowed to drain off and placed in:

2. Alcohol 80 percent for 6 hours, then
   Alcohol 95 percent for 2 hours.

From this directly into

3. Water.............................................2000
   Acetate of potash.................... ............ 200
   Glycerin ....... .................................... 400

for permanent preservation in a dark place.

Further details for the preservation of whole organs, etc., should be studied in the original article (C. Kaiserling, Virch. Arch. Bd. 147s, 389).

The results thus obtained are certainly wonderful, and promise to be one of the greatest aids to teaching of this generation.—*American Medico-Surgical Bulletin.*

